# Controlling the Two-Photon-Induced Photon Cascade Emission in a Gd^3+^/Tb^3+^-Codoped Glass for Multicolor Display

**DOI:** 10.1038/srep21091

**Published:** 2016-02-22

**Authors:** Mao-Hui Yuan, Hai-Hua Fan, Hui Li, Sheng Lan, Shao-Long Tie, Zhong-Min Yang

**Affiliations:** 1 Guangdong Provincial Key Laboratory of Nanophotonic Functional Materials and Devices, School of Information and Optoelectronic Science and Engineering, South China Normal University, Guangzhou 510006, China; 2 School of Chemistry and Environment, South China Normal University, Guangzhou 510006, China; 3 MOE Key Laboratory of Specially Functional Materials, Institute of Optical Communication Materials, South China University of Technology, Guangzhou 510640, China

## Abstract

We reported the first observation of the two-photon-induced quantum cutting phenomenon in a Gd^3+^/Tb^3+^-codoped glass in which two photons at ~400 nm are simultaneously absorbed, leading to the cascade emission of three photons in the visible spectral region. The two-photon absorption induced by femtosecond laser pulses allows the excitation of the energy states in Gd^3+^ which are inactive for single-photon excitation and enables the observation of many new electric transitions which are invisible in the single-photon-induced luminescence. The competition between the two-photon-induced photon cascade emission and the single-photon-induced emission was manipulated to control the luminescence color of the glass. We demonstrated the change of the luminescence color from red to yellow and eventually to green by varying either the excitation wavelength or the excitation power density.

The phenomenon of photon cascade emission or the so called “quantum cutting”, in which a photon of high energy is absorbed and converted to two or more photons with lower energies, has been studied intensively in the past few decades because of its potential applications in mercury-free lamps and plasma display panels^1^,^2^,^3^,^4^,^5^,^6^,^7^,^8^,^9^. In recent years, this phenomenon has drawn great attention in the research and development of high-efficiency solar cells because it can significantly improve the conversion efficiency of photon to electricity and reduce heat generation^10^,^11^,^12^,^13^. Owing to their unique energy states, rare-earth ions, especially the lanthanide ions, are considered as promising candidates not only for photon up-conversion but also for photon down-conversion^13^,^14^,^15^,^16^,^17^,^18^,^19^,^20^,^21^,^22^. For example, solid state full color display^23^ has been demonstrated by exploiting the photon up-conversion in three lanthanide ions of Pr^3+^, Er^3+^, and Tm^3+^. In addition, the lanthanide ions have exhibited fascinating luminescent properties such as intense narrow-band emission, high conversion efficiency, broad emission peaks, much different lifetimes, and good thermal stability^8^,^21^,^24^,^25^,^26^,^27^. Therefore, rare-earth-ion-doped materials have been widely studied and exhibited potential application in the fields of illumination, imaging, display, solar cells, and medical radiology because such materials can be fabricated at a low cost and in large quantities^23^,^24^,^25^,^26^,^27^,^28^,^29^,^30^,^31^,^32^,^33^,^34^,^35^,^36^,^37^,^38^,^39^,^40^,^41^,^42^,^43^,^44^.

In rare-earth-ion-doped materials, Tb^3+^-doped glasses have been the focus of many studies because of their high luminescence efficiency at around 550 nm which is convenient for direct coupling with silicon detectors^45^. More interestingly, it has been shown that the luminescence can be further enhanced by adding Gd^3+^ into Tb^3+^-doped glasses because of the energy transfer (ET) from Gd^3+^ to Tb^3+^, as schematically shown in Fig. 1. In fact, the ET between Gd^3+^ and Tb^3+^ has been extensively investigated in many other different host materials^46^,^47^,^48^,^49^,^50^. Although electrons can be generated in Gd^3+^, the luminescence from Gd^3+^/Tb^3+^-codoped glasses arises mainly from the transitions from the level ^5^D_4_ to the levels ^7^F_0–6_ in Tb^3+^ which give rise to four emission bands in the visible light region^46^,^47^,^48^,^49^,^50^. In Fig. 1, it is noticed that the levels ^6^G_J_ in Gd^3+^ are located well above the high-energy levels in Tb^3+^ (^5^K_7_ etc.) while the levels ^6^D_J_, ^6^I_J_, and ^6^P_J_ in Gd^3+^ have similar energies with some energy levels in Tb^3+^. If the population of the levels ^6^G_J_ in Gd^3+^ is induced, one can expect the transitions of electrons to the low-energy levels (^6^D_J_, ^6^I_J_, and ^6^P_J_) of Gd^3+^, the ET of electrons from Gd^3+^ to Tb^3+^, the transitions of electrons to the level ^5^D_4_ and finally to the levels ^7^F_0–6_. Such a cascade transition process may result in the cascade emission of photons with different energies. In practice, the population of the levels ^6^G_J_ in Gd^3+^ can be realized by using femtosecond (fs) laser light at ~400 nm through two-photon-induced absorption (TPA). The high peak power and wide linewidth of fs laser light are highly suitable for effectively exciting the levels ^6^G_J_ in Gd^3+^. Actually, fs laser light at 800 nm has been used to excite the three-photon-induced luminescence in rare-earth-ion-doped glasses^37^. When fs laser light at 400 nm is used to excite the levels ^6^G_J_ in Gd^3+^, the level ^5^D_3_ in Tb^3+^ with a wavenumber of ~26336 cm^−1^ (corresponding to a wavelength of ~381 nm) can also be populated through Rabi oscillation or phonon-assisted transition^51^, leading to the conventional emission from Tb^3+^. For excitation wavelengths (*λ*_ex_) shorter than 400 nm, the population probability for the levels ^6^G_J_ is reduced while that for the level ^5^D_3_ is increased. It implies the existence of a competition between the cascade emission and conventional emission that depends strongly on *λ*_ex_. On the other hand, the population of the level ^5^D_3_, which is caused mainly by Rabi oscillation^51^, will exhibit a strong dependence on the excitation power density (*P*_ex_). Therefore, it is expected that one can manipulate the competition between the cascade emission and conventional emission and thus control the luminescence color by varying *λ*_ex_ or *P*_ex_, exploring its applications in color display^52^.

## Results and Discussion

The proposed scheme was examined by using different glasses codoped with Gd^3+^ and Tb^3+^ and the dependence of luminescence color on *λ*_ex_ and *P*_ex_ was found to be a popular phenomenon (details in Supplementary Information, see Fig. S1). However, this behavior also exhibits a dependence on the concentration of Gd^3+^ and glass matrix. While the concentration of Gd^3+^ determines the photon cascade emission, the glass matrix affects the phonon-assisted processes such as the nonradiative decay or the relaxation of electrons. In this work, we show a pronounced phenomenon observed in a silicate glass with a composition of 56SiO_2_-10Al_2_O_3_-12Li_2_O-20Gd_2_O_3_-2Tb_2_O_3_ (mol%). The ET from Gd^3+^ to Tb^3+^ was also observed in this glass (details in Supplementary Information, see Section 4).

In order to see the *λ*_ex_-dependent competition, we varied *λ*_ex_ from 375 to 405 nm and examined the luminescence of the glass. For *λ*_ex_ ≤ 390 nm, the luminescence always appeared to be green. However, the luminescence was changed from green to yellow when *λ*_ex_ was slightly shifted from 390 to 392 nm. More surprisingly, the luminescence turned to be red when *λ*_ex_ was further shifted to 394 nm. A comparison of the emission spectra under different *λ*_ex_ of 390, 392 and 394 nm is presented in Fig. 2. The photos for the excitation spot are shown in the insets. It can be seen that the emission spectrum at *λ*_ex_ = 390 nm is dominated by the emission band at ~540 nm which corresponds to green color. For *λ*_ex_ = 392 nm, the relative intensities of the emission bands at ~580 nm and ~622 nm, which correspond to yellow and red colors, increase rapidly. A close inspection reveals that the peak of the emission band at ~622 nm is blue-shifted to ~613 nm. In addition, two new emission bands emerge at ~654 nm and ~704 nm, contributing to red color. For *λ*_ex_ = 394 nm, the intensity of the emission band at ~613 nm exceeds that of the emission band at ~550 nm and the relative intensities of the emission bands at ~654 and 704 nm are further increased, turning the color of the luminescence into red. In previous reports, the luminescence of Tb^3+^-doped and Gd^3+^/Tb^3+^-codoped glasses originates mainly from the electronic transitions from the level ^5^D_4_ to the levels ^7^F_0–6_ and appears to be green. There is no report on the observation of yellow or red luminescence. Here, it is interesting that the color change in the luminescence occurs in a narrow wavelength region of 390−394 nm, which is comparable to the linewidth of the fs laser pulses (~4.0 nm).

In order to understand the underlying physical mechanism, we examined the evolution of the emission spectrum and the luminescence color with increasing *P*_ex_ for different *λ*_ex_, as shown in Fig. 3a–c. The dependence of the luminescence intensities for different emission bands on *P*_ex_ was also extracted, as shown in Fig. 3d–f. For *λ*_ex_ = 390 nm, the luminescence color appeared to be green and remained unchanged with increasing *P*_ex_. It can be seen that the intensities of the four major emission bands, which are centered at 488, 544, 585, and 622 nm, increased almost with the same rate. Consequently, the emission spectrum remained nearly unchanged except the absolute intensity. The fitting of the *P*_ex_ dependence of the luminescence intensity gives nearly the same slope of ~1.0 for all the emission bands, indicating that the emission is governed by single-photon process. The situation was changed for *λ*_ex_ = 392 nm. Two new emission bands emerged at 654 and 704 nm and the emission band peaking originally at 622 nm was blue-shifted to 613 nm. The electronic transitions related to the new emission bands (details in Supplementary Information, see Table I), which are attributed to the transitions between the high-energy levels of Gd^3+^, were previously observed in other materials^53^,^54^ doped with Gd^3+^ under single-photon excitation. For comparison, the glasses doped with only Gd^3+^ or Tb^3+^ were also examined under the same condition (details in Supplementary Information, see Figs S3 and S5). The slopes for the emissions bands at 488, 544, and 585 nm remained unchanged while smaller slopes were observed for the two new emission bands and the emission band at 613 nm. The change in the emission spectrum was negligible and the luminescence color remained to be yellow with increasing *P*_ex_. For *λ*_ex_ = 394 nm, the dependence of the emission spectrum on *P*_ex_ became significant. It is noticed that the intensities of the emission bands at 488 and 544 nm increased in greater rates and the center of the excitation spot became yellow at high *P*_ex_, as evidenced in the larger slopes of ~1.40. This behavior indicates that two-photon process has been involved in the emission process.

As mentioned above, the effective population of the levels ^6^G_J_ can be realized by simultaneously absorbing two photons at 400 nm. For *λ*_ex_ = 390 nm, the population of the levels ^6^G_J_ through TPA can be neglected because of the large energy mismatch. As *λ*_ex_ is red-shifted toward 400 nm, an increase in the population probability is expected. In Fig. 4, we present the evolution of the emission spectrum and the luminescence color with increasing *P*_ex_ measured for *λ*_ex_ = 400 and 405 nm. For *λ*_ex_ = 400 nm, the emission spectrum was similar to that observed at *λ*_ex_ = 394 nm. However, it is noticed that the intensity of the emission band at 613 nm became stronger than that of the emission band at 544 nm and the luminescence appeared to be red at low *P*_ex_. At high *P*_ex_, the intensity of the latter exceeded that of the former, changing the luminescence color to green, as shown in the inset of Fig. 4a. This behavior is also reflected in the *P*_ex_ dependence of the luminescence intensity shown in Fig. 4c. The slopes for the emission bands at 488 and 544 nm obtained by fitting the experimental data (1.59 and 1.64) were much larger than those for the emission bands at 590, 613, 654, and 704 nm whose slopes are reduced by nonradiative decay (details in Supplementary Information, see Fig. S6). For *λ*_ex_ = 405 nm, the emission spectrum was changed remarkably because of the emergence of many new emission bands, as shown in Fig. 4b. In this case, the red luminescence observed at low *P*_ex_ evolved gradually into green one with increasing *P*_ex_, similar to that observed at *λ*_ex_ = 400 nm. In comparison, the slopes for the emission bands at 488 and 544 nm were reduced slightly to ~1.50 because the TPA process at *λ*_ex_ = 405 nm is not as efficient as that at *λ*_ex_ = 400 nm.

Based on the theory of colorimetry (details in the Supplementary Information, see Section 7), one can easily deduce the chromaticity coordinates for the luminescence observed under different excitation conditions and the results are shown in Fig. 5. From the Fig. 5a, under low *P*_ex_, we can see that the chromaticity coordinates for *λ*_ex_ = 390, 392, and 394 nm appear in the green, yellow and red regions, respectively. In all cases, a shift of the chromaticity coordinate with increasing *P*_ex_ is found and it becomes larger for longer *λ*_ex_. The largest shift of the chromaticity coordinate is observed at *λ*_ex_ = 400 nm, as shown in Fig. 5b. In this case, the luminescence color is changed from red to yellow and eventually to green with increasing *P*_ex_.

**Figure 1:**
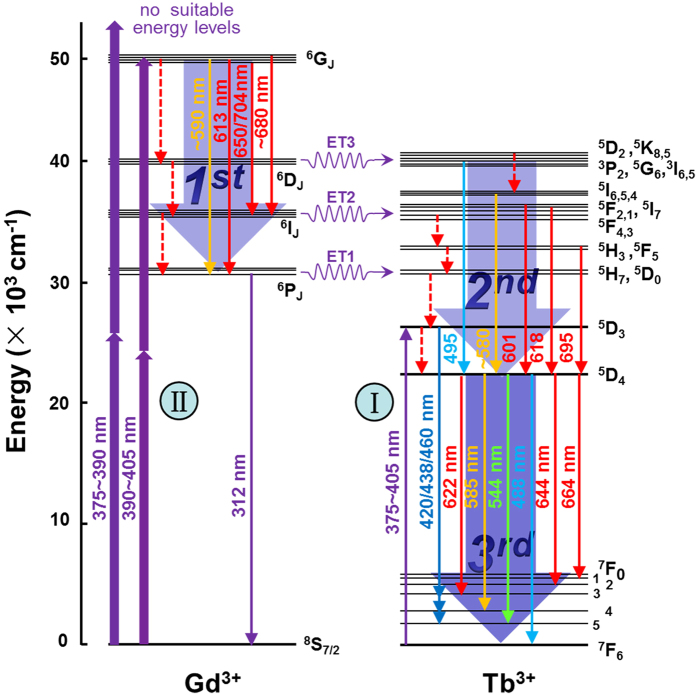
Energy level diagram of Gd^3+^ and Tb^3+^ in which the two-photon-induced absorption, the photon cascade emission 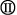, and the single photon absorption assisted by Rabi oscillation or phonons 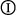, and the possible electronic transitions between the energy levels are illustrated.

**Figure 2:**
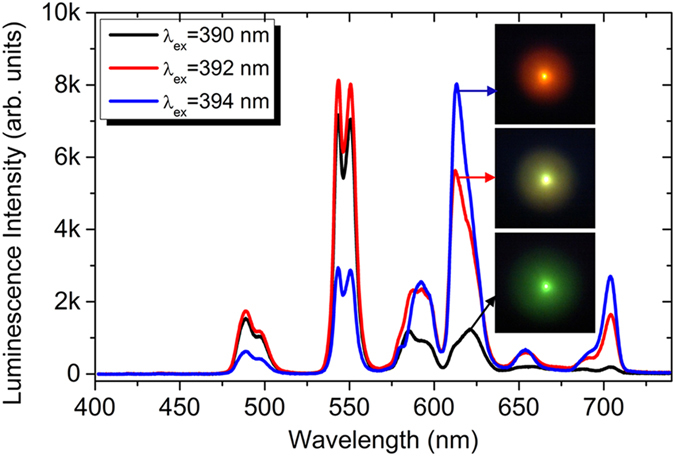
Comparison of the emission spectra of the glass under different *λ*_ex_ of 390, 392, and 394 nm. The insets show the photos of the excitation spots and the luminescence colors.

**Figure 3:**
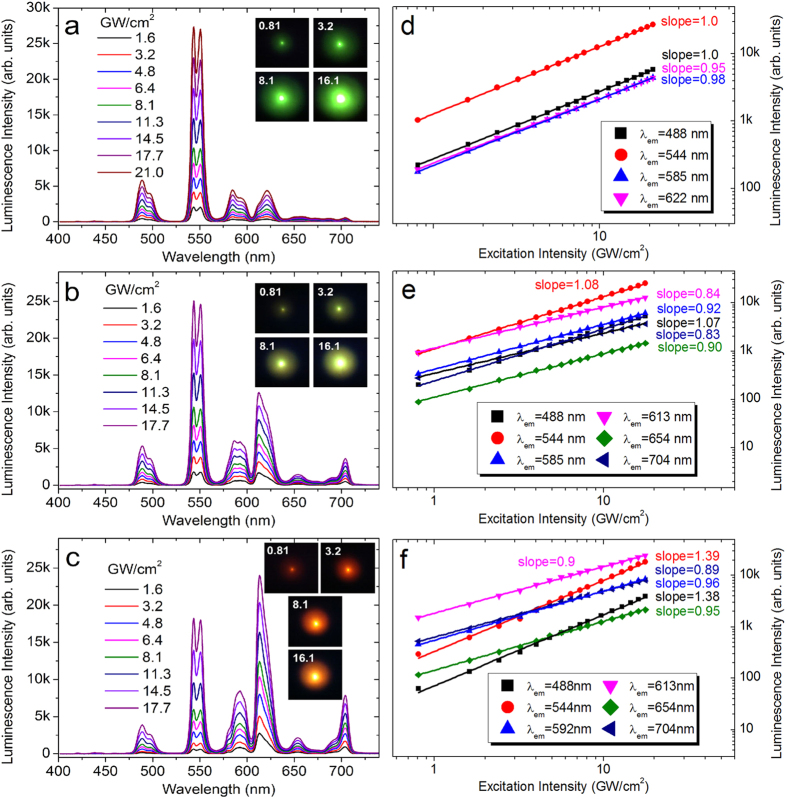
Evolution of the emission spectrum and the luminescence color of the glass with increasing *P*_ex_ under different *λ*_ex_ of (**a**) 390 nm, (**b**) 392 nm, and (**c**) 394 nm. The dependence of the luminescence intensities for different emission bands on *P*_ex_ and the fitting for the experimental data are presented in (**d**–**f**) for *λ*_ex_ of 390, 392, and 394 nm, respectively.

**Figure 4:**
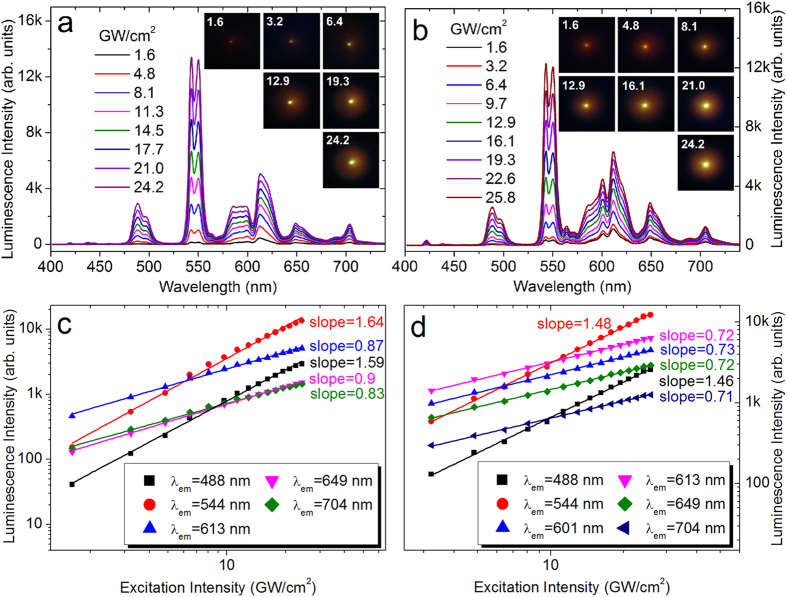
Evolution of the emission spectrum and the luminescence color of the glass with increasing *P*_ex_ under different *λ*_ex_ of (**a**) 400 nm and (**b**) 405 nm. The dependence of the luminescence intensities for different emission bands on *P*_ex_ and the fitting for the experimental data are presented in (**c**,**d**) for *λ*_ex_ of 400 and 405 nm, respectively.

**Figure 5:**
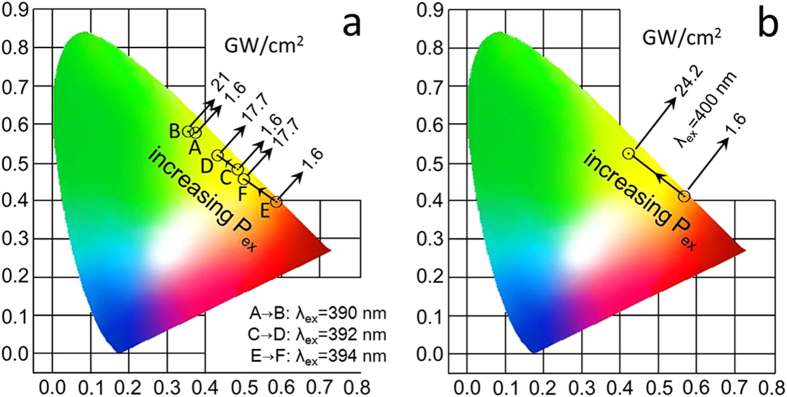
CIE chromaticity coordinates for the luminescence of the glass under different *λ*_ex_ and *P*_ex_. (**a**) *λ*_ex_ = 390, 392, and 394 nm and (**b**) *λ*_ex_ = 400 nm.

**Figure 6:**
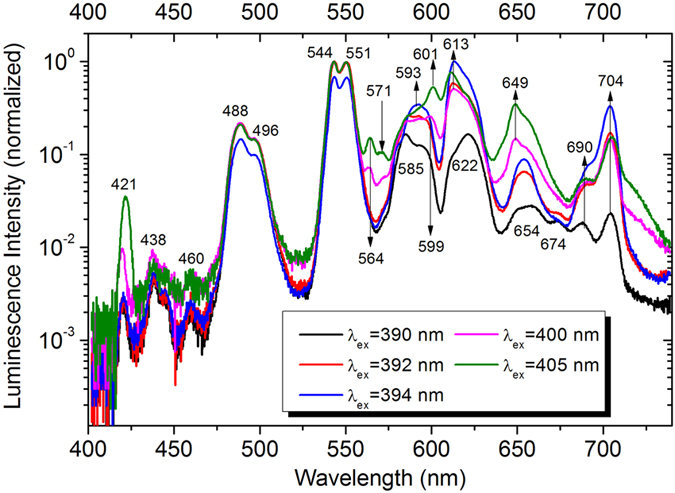
Comparison of the emission spectra of the glass plotted in a logarithmic coordinate under different *λ*_ex_ of 390, 392, 394, 400, and 405 nm. The new emission bands observed in the photon cascade emission are indicated by arrows.

When the levels ^6^G_J_ in Gd^3+^ are effectively populated, the photon cascade emission is expected to dominate the emission process. It is clearly reflected in the new emission bands which correspond to the photons emitted in the cascade transition of electrons between the energy levels of Gd^3+^ and Tb^3+^. In Fig. 6, we present a comparison of the emission spectra obtained at different *λ*_ex_ of 390, 392, 394, 400, and 405 nm in a logarithmic coordinate where the new emission bands originating from the photon cascade emission can be readily identified at *λ*_ex_ = 400 and 405 nm and indicated by arrows. In most cases, the cascade emission of three photons is observed, as shown in Fig. 1. The first photon is generated by the transition of electrons between the levels of ^6^G_J_ and ^6^I_J_ (or ^6^P_J_) in Gd^3+^. The wavelengths of the emission photons range from 571 to 704 nm, contributing yellow or red color. The transition from the levels ^6^G_J_ to ^6^D_J_ in Gd^3+^ is thought to be nonradiative^53^,^54^. The emission of the first photon is followed by an ET process of electrons from Gd^3+^ to Tb^3+^. Then, the emission of the second photon occurs through the transitions of electrons between the levels ^5^H_3_, ^5^F_4,2,1_, ^5^I_6,4_, ^5^K_8_ and the level ^5^D_3_ or ^5^D_4_. During this process, the wavelengths of the emission photons cover a broad wavelength range of 495 to 690 nm, contributing mainly yellow and red colors. The emission of the last photon occurs mainly between the levels ^5^D_4_ (^5^D_3_) and ^7^F_6,5,4,3_, contributing to green color. Although the intensities are quite weak, one can identify the new emission bands at 421, 438, and 460 which can be assigned to the transitions between the levels ^5^D_3_ and ^7^F_5,4,3_.

Having understood the photon cascade emission, one can easily understand the luminescence color change induced by varying *λ*_ex_ and *P*_ex_. For *λ*_ex_ < 392 nm, the levels ^6^G_J_ are not populated effectively and the photon cascade emission is not initiated. In this case, the population of the levels ^5^D_3_ and ^5^D_4_ in Tb^3+^ through Rabi oscillation or phonon assistance is dominant, giving rise to the four emission bands and green luminescence. For *λ*_ex_ > 392 nm, the electrons begin to occupy the levels ^6^G_J_, initiating the photon cascade emission which competes with the conventional emission. When the emission process becomes dominated by the photon cascade emission, the luminescence appears to be yellow or red. With increasing *P*_ex_, the population of the level ^5^D_3_ becomes significant because of two reasons. First, the population probability due to Rabi oscillation increases with increasing *P*_ex_. Second, more electrons relax from the levels ^6^G_J_ to the level ^5^D_3_ after emitting two photons. Therefore, a rapid increase in the intensities of the emission bands at 488 and 544 nm is observed, turning the luminescence into yellow and green at high *P*_ex_. In our case, it was found that the efficiency of the two-photon-induced transitions and ET process from Gd^3+^ to Tb^3+^ depends strongly on the concentrations of Gd^3+^ and Tb^3+^ because it is proportional to the inverse sixth power of the distance between the two types of ions (details in the Supplementary Information, see Section 5). In this work, we have compared the tunable range of the luminescence color by varying *λ*_ex_ and *P*_ex_ for several glass samples with different concentrations of Gd^3+^ and found that the best performance was achieved in the glass with the largest concentration of Gd^3+^.

## Conclusion

In conclusion, we have observed the two-photon-induced photon cascade emission in a glass codoped with Gd^3+^ and Tb^3+^ by using fs laser pulses. By varying either *λ*_ex_ or *P*_ex_, we have demonstrated the manipulation of the luminescence color through the competition between the photon cascade emission and conventional emission. Many new emission bands, which are not usually observed in the traditional luminescence of Tb^3+^-doped or Gd^3+^/Tb^3+^-codoped materials have been clearly revealed in the emission spectrum when the photon cascade emission is dominant. More importantly, the dependence of the luminescence on both *λ*_ex_ and *P*_ex_ implies potential applications in laser-induced color display.

## Methods

### Materials Preparation

The silicate glass was prepared through melting the mixture of analytical reagents of SiO_2_, Al(OH)_3_, Li_2_CO_3_, Gd_2_O_3_, and Tb_4_O_7_ at 1600 °C for 30 minutes. The melt was then poured onto a preheated (200 °C) stainless steel plate and annealed at 500 °C for 2 hours. The synthetized glass was cut into 10 × 10 × 1 mm^3^ sheets and polished for optical measurements.

### Photonluminescence Measurements

In our experiments, the fs laser light with a repetition rate of 76 MHz and a pulse duration of ~130 fs delivered by a Ti: sapphire oscillator (Mira 900S, Coherent) was introduced into a harmonic generator (Harmonics 9300, Coherent) and the output light with a tunable wavelength from 375 to 405 nm was employed to excite the glass. The excitation intensity was characterized by the peak power density of the fs laser pulses. It was introduced into an inverted microscope (Observer A1, Zeiss) and focused on the glass by using a 60 × objective lens (NA = 0.85). The diameter of the excitation spot was estimated to be ~4.0 μm. The luminescence generated by the glass was collected by the same objective lens and delivered to a spectrometer (SR-500I-B1, Andor) equipped with a charge-coupled device (CCD) (DU970N, Andor) for analysis. The photos of the excitation spot were taken by using a camera from the eyepiece of the microscope.

## Additional Information

**How to cite this article**: Yuan, M.-H. *et al.* Controlling the Two-Photon-Induced Photon Cascade Emission in a Gd^3+^/Tb^3+^-Codoped Glass for Multicolor Display. *Sci. Rep.* **6**, 21091; doi: 10.1038/srep21091 (2016).

## Supplementary Material

Supplementary Information
