# Supplementary material for: Controlling the Two-Photon-Induced Photon Cascade Emission in a Gd3+/Tb3+-Codoped Glass for Multicolor Display
Source: Sci Rep. 2016 Feb 22;6:21091. doi: 10.1038/srep21091 (PMC4761965; doi:10.1038/srep21091)
Supplement: Supplementary Information [file srep21091-s1.pdf]

Supplementary Information for "Controlling the Two-Photon-Induced Photon Cascade Emission in a Gd^{3+}/Tb^{3+}-Codoped Glass for Multicolor Display"

Mao-Hui Yuan, Hai-Hua Fan, Hui Li, Sheng Lan*, Shao-Long Tie*, and Zhong-Min Yang

1. Dependence of luminescence color on excitation wavelength and excitation power observed in other Gd^{3+}/Tb^{3+}-codoped glasses

In this work, we proposed a scenario for controlling the luminescence color of a Gd^{3+}/Tb^{3+}-codoped glass by exploiting the competition between the two-photon-induced cascade emission and the conventional emission, as schematically shown in Fig. 1. In principle, the proposal should work for any glasses codoped with Gd^{3+} and Tb^{3+}. In the following, we show the similar behavior observed in a boron aluminate glass with a composition $42CaO$-$15Al_2O_3$-$39.5B_2O_3$-$3Gd_2O_3$-$0.5Tb_2O_3$ (mol%) in which the concentration of Gd^{3+} is much smaller than that in the silicate glass. In Figs. S1(a), S1(b), S1(c) and S1(d), we present the evolution of the emission spectrum with increasing excitation power (P_{ex}) measured at different excitation wavelength (λ_{ex}) of 394, 397, 400, and 405 nm, respectively. For $\lambda_{ex} < 394$ nm, the luminescence appeared to be green and no change in the luminescence color was observed with increasing P_{ex}. When λ_{ex} was shifted to 397 nm, the luminescence appeared to be red at low P_{ex} and it was changed to green with increasing P_{ex}. The dependence of the luminescence color on P_{ex} becomes more significant at $\lambda_{ex} = 405$ nm. It means that the luminescence color of the boron aluminate glass can also be controlled by varying λ_{ex} or P_{ex}, as manifested in the chromaticity coordinates shown in Fig. S2. For each λ_{ex}, the dependences of the luminescence intensities for different emission bands on P_{ex} are extracted and presented in Figs. S1(e), S1(f), S1(g) and S1(h). Similar to the phenomenon observed in the silicate glass, the slopes for all emission bands are close to 1.0 for $\lambda_{ex} < 394$ nm when the two-photon-induced cascade emission is not initiated. However, the effect is expected to depend on the concentration of Gd^{3+} and glass matrix. While the concentration of Gd^{3+} determines the photon cascade emission, the glass matrix affects the phonon-assisted processes such as the nonradiative decay or the relaxation of electrons. We have examined the proposed scheme by using different glasses codoped with Gd^{3+} and Tb^{3+} and confirmed the change of luminescence color with different λ_{ex} or P_{ex}. So far, the most pronounced phenomenon was observed in a silicate glass with a composition of $56SiO_2$-$10Al_2O_3$-$12Li_2O$-$20Gd_2O_3$-$2Tb_2O_3$ (mol%), as presented

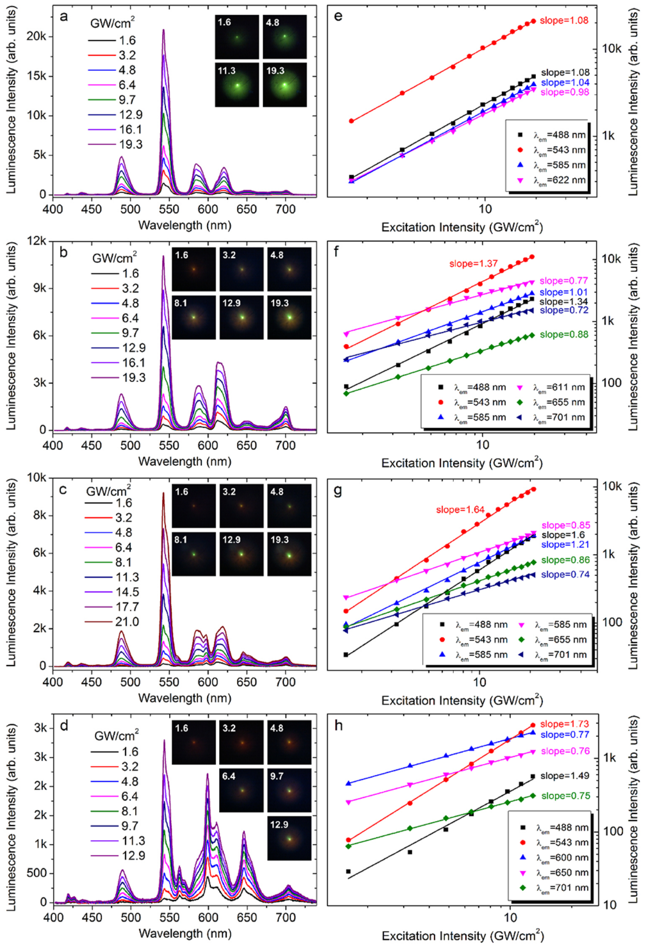

FIG. S1. Evolution of the emission spectrum and the luminescence color of the boron aluminate glass ($42CaO$-$15Al_2O_3$-$39.5B_2O_3$-$3Gd_2O_3$-$0.5Tb_2O_3$ (mol%)) with increasing P_{ex} under different λ_{ex} of (a) 394 nm, (b) 397 nm, (c) 400 nm, and (d) 405 nm. The dependence of the luminescence intensities for different emission bands on P_{ex} and the fitting for the experimental data are presented in (e), (f), (g), and (h) for different λ_{ex} of 394, 397, 400, and 405 nm, respectively. The insets show the photos of the excitation spots and the luminescence colors.

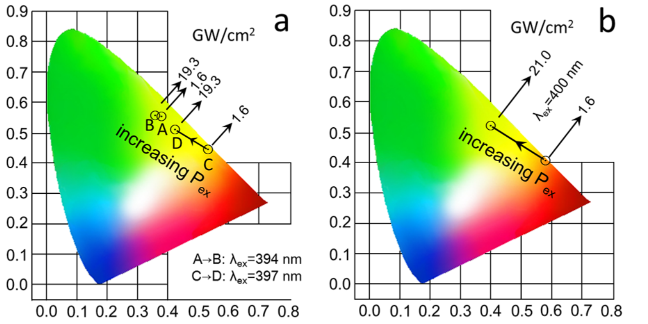

FIG. S2. CIE chromaticity coordinates for the luminescence of the boron aluminate glass ($42CaO$-$15Al_2O_3$-$39.5B_2O_3$-$3Gd_2O_3$-$0.5Tb_2O_3$ (mol%)) under different λ_{ex} and P_{ex}. (a) $\lambda_{ex} = 394$, 397 nm and (b) $\lambda_{ex} = 400$ nm.

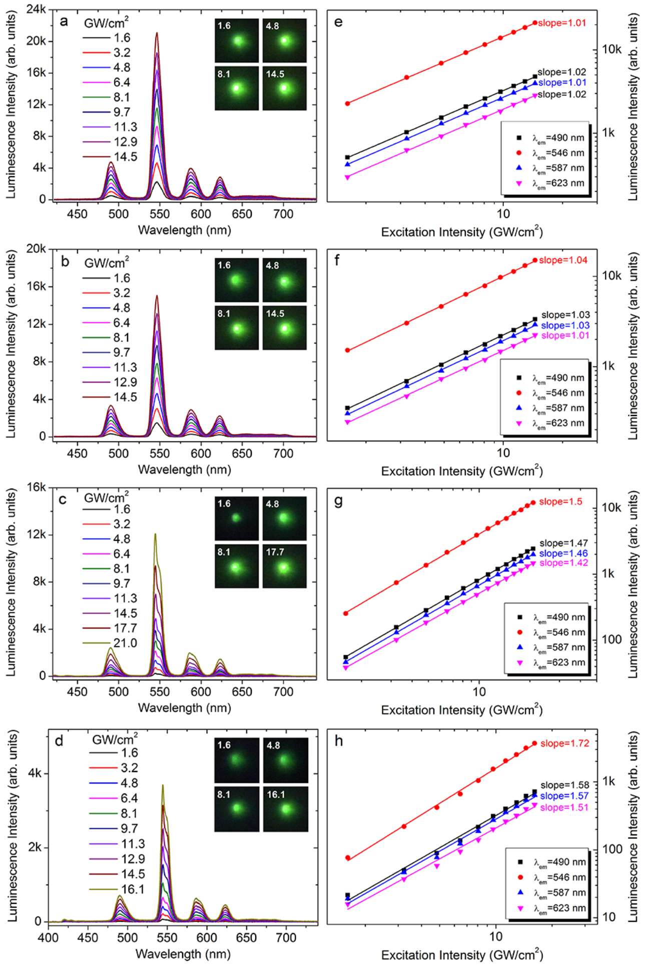

FIG. S3. Evolution of the emission spectrum and the luminescence color of the boron aluminate glass ($45CaO$-$15Al_2O_3$-$39.5B_2O_3$-$0.5Tb_2O_3$ (mol%)) with increasing P_{ex} under different λ_{ex} of (a) 392 nm, (b) 394 nm, (c) 400 nm, and (d) 405 nm. The dependence of the luminescence intensities for different emission bands on P_{ex} and the fitting for the experimental data are presented in (e), (f), (g), and (h) for different λ_{ex} of 392, 394, 400, and 405 nm, respectively.

in the main text. For $\lambda_{ex} \geq 397$ nm, large slopes close to 2.0 are observed for the emission bands centered at 488 and 543 nm because of the initiation of the two-photon-induced cascade emission, leading to the change of the luminescence color. Since the energies levels of rare-earth ions may be slightly different in different glass matrices [1], the λ_{ex} at which the change in the luminescence color occurs are slightly different. The luminescence color change occurs at 394 and 397 nm for the silicate glass and the boron aluminate glass, respectively. We also examined boron aluminate glasses with smaller concentrations of Gd^{3+} (<3%). It was found that the phenomenon became less obvious and disappeared completely in the glass doped only with Tb^{3+} (see Fig. S3). Another Gd^{3+}/Tb^{3+}-codoped glass we have examined is $65SiO_2$-$13BaO$-$5K_2O$-$5Na_2O$-$1Al_2O_3$-$5Li_2O$-$5Gd_2O_3$-$1Tb_2O_3$ (mol%). The photos of the excitation spots showing the dependence of the luminescence color

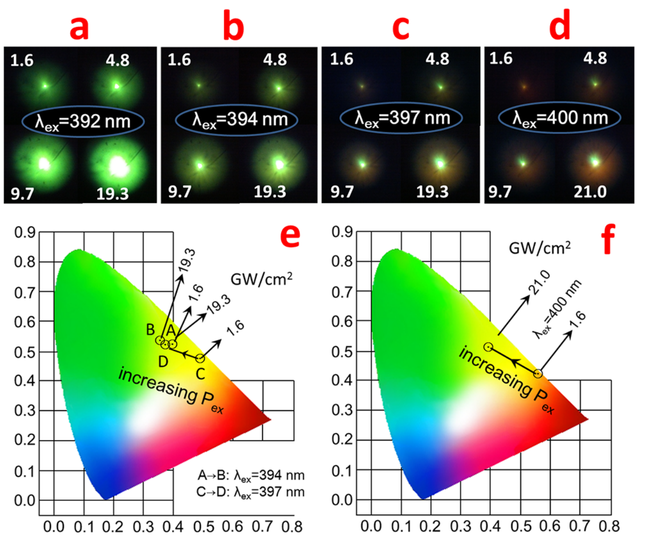

FIG. S4. Evolution of the luminescence color of the silicate glass ($65SiO_2$-$13BaO$-$5K_2O$-$5Na_2O$-$1Al_2O_3$-$5Li_2O$-$5Gd_2O_3$-$1Tb_2O_3$ (mol%)) with increasing P_{ex} under different λ_{ex} of (a) 392 nm, (b) 394 nm, (c) 397 nm, and (d) 400 nm. The CIE chromaticity coordinates for the luminescence of the silicate glass under different P_{ex} and λ_{ex} are shown in (e) and (f).

on λ_{ex} and P_{ex} are presented in Figs. S4(a)-S4(d). The tuning of the chromaticity coordinates by varying λ_{ex} and P_{ex} are shown in Fig. S4(e) and S4(f). Based on the experimental results mentioned above, we think that the proposed scheme can be realized in different Gd^{3+}/Tb^{3+}-codoped glasses and the luminescence color change can be optimized by choosing suitable glass matrix and doping levels of Gd^{3+} and Tb^{3+}.

2. Dependence of luminescence intensity on excitation power for energy levels with nonradiative decay

From the energy diagram of Gd^{3+} shown in Fig. 1, it can be seen that the energy separation between the levels ^6P_J and $^8S_{7/2}$ is $\sim$32000 cm^{-1}, corresponding to a wavelength of $\sim$312 nm. As a result, the linear absorption of Gd^{3+} at 400 nm is negligible and that of the silicate glass is quite small because of the low concentration of Tb^{3+}, as shown in our previous work [2]. Therefore, any emissions related with Gd^{3+}, such as the emission bands centered at 613, 654, and 704 nm, must be induced at least by two-photon absorption (TPA). It is expected that a slope of 2.0 will be observed in the dependence of the luminescence intensity on P_{ex} when the two-photon-induced cascade emission become dominant for $\lambda_{ex} >$ 394 nm. However, it is noticed that the dependences of the luminescence intensities for the emission bands centered at 613, 654, and 704 nm on P_{ex}, which are shown in Figs. 3 and 4, exhibits slopes even smaller than 1.0. We also examined the two-photon-induced cascade emission process in a boron aluminate glass ($43CaO$-$15Al_2O_3$-$39B_2O_3$-$3Gd_2O_3$ (mol%)) doped only with Gd^{3+} and the

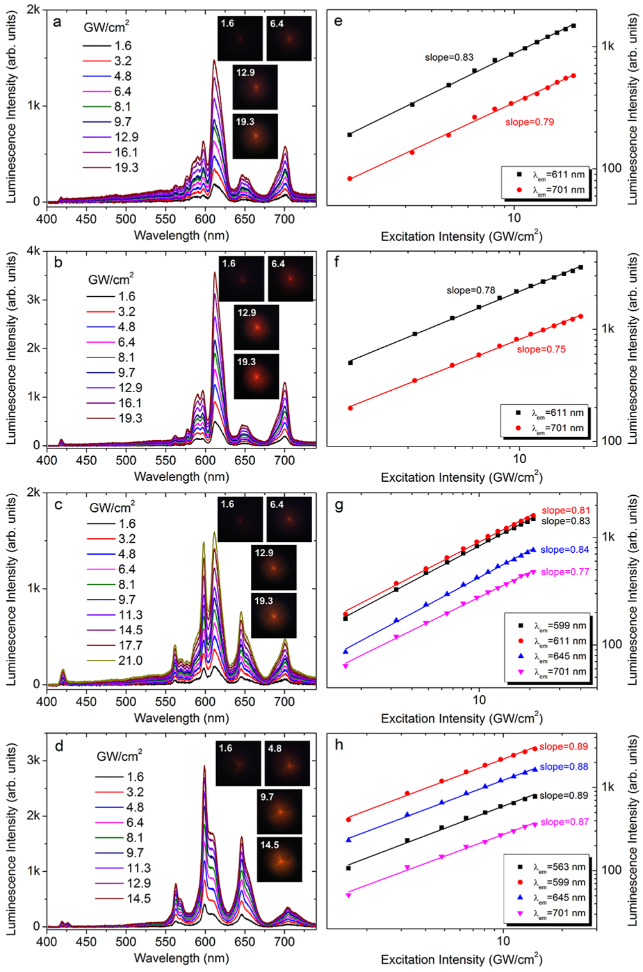

FIG. S5. Evolution of the emission spectrum and the luminescence color of the glass ($42.5CaO$-$15Al_2O_3$-$39.5B_2O_3$-$3Gd_2O_3$ (mol%)) with increasing P_{ex} under different λ_{ex} of (a) 394 nm, (b) 397 nm, (c) 400 nm, and (d) 405 nm. The dependence of the luminescence intensities for different emission bands on P_{ex} and the fitting for the experimental data are presented in (e), (f), (g), and (h) for different λ_{ex} of 394, 397, 400, and 405 nm, respectively.

results are presented in Fig. S5. Except the emission band centered at ∼312 nm ($^6P_J \rightarrow {}^6S_{7/2}$) which is out of the detection region, it can be seen that the slopes for all the detectable emission bands are smaller than 1.0. This behavior is different from the conventional two-photon-induced luminescence process which generally shows a slope of ∼2.0. Previously, it was revealed that the upconversion luminescence excited by sequential absorption of n photons may exhibit a dependence on excitation intensity P in the range of P^n to P^1 [3]. The two limits are identified as the cases of infinitely small and infinitely large upconversion rate. If the transition from the ground state to a high-energy state is achieved by simultaneous absorption of n photons to an intermediate state followed by the simultaneous absorption of m photons, it is expected that the excitation intensity dependence of the upconversion luminescence will exhibit a slope between n and $m + n$, which is considered as the index of the corresponding nonlinear process. This behavior has been experimentally demonstrated by us in GaN/InGaN multiple quantum wells [4]. However, there is no such intermediate state in Gd^{3+} and the physical origin for small slopes is the existence of nonradiative decay in the high-energy levels involved in the electronic transitions, as demonstrated in the following.

In Fig. S6(a), we use a simple three-level model to explain why the slope extracted from the P_{ex} dependence of luminescence intensity becomes much smaller than 2.0 in the presence of nonradiative decay. Supposing that the transition from the level E_1 to the level E_2 is induced by TPA, let's see what will happen when the transition of electrons from the level E_2 to the level E_3 occurs either radiatively or nonradiatively, similar to the transitions from the level ^6G_J to the levels ^6I_J and ^6P_J in Gd^{3+}. While the radiative transition is completed by the emission of photons, the nonradiative transition is accompanied by the emission of phonons. Actually, the photon cascade emission induced by single-photon absorption was observed previously in a fluoride glass doped with Gd^{3+} [5]. The luminescence intensities resulting from the transitions from the level ^6G_J to the levels ^6I_J and ^6P_J were found to be much weaker than that resulting from the transition from the level ^6P_J to the level $^6S_{7/2}$. It was ascribed to the existence of nonradiative decay in the transitions from the level ^6G_J to the levels ^6I_J and ^6P_J. In general, the nonradiative transition is mediated by multiphonon process whose rate is proportional to $(1+\langle n\rangle^P)$, where $\langle n\rangle$ is the phonon density which increases exponentially with the temperature of the glass matrix. The higher the temperature is, the larger the multiphonon relaxation rate is [6]. Therefore, it is expected that the nonradiative decay rate increases

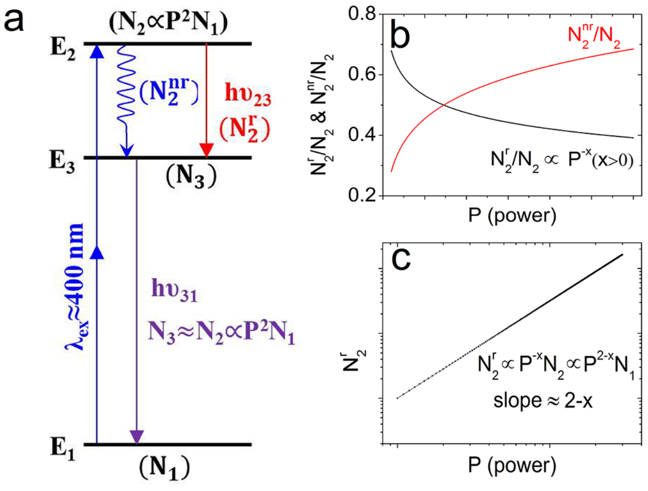

FIG. S6. (a) Energy diagram showing the two-photon-induced absorption followed by radiative and nonradiative decays of electrons. (b) Dependence of the radiative and nonradiative decays of population density on P_{ex}. (c) P_{ex} dependence of the radiative decay of population density (i.e., luminescence intensity) in the presence of nonradiative decay. The (b) and (c) are theoretical curves.

TABLE I. Possible transitions between the energy levels in Gd^{3+} and Tb^{3+} that may involved in the two-photon-induced photon cascade emission and the corresponding emission wavelengths. Most of them can be identified in the emission spectra with λ_{ex} = 400 and 405 nm (see Fig. 6).

$Gd^{3+}(nm)$	$Tb^{3+}(nm)$
$^6G_{13/2} \to {}^6I_{15/2} = 688$	$^5F_5 \to {}^5D_4 = 695$
$^6G_{13/2} \to {}^6I_{13/2} = 687$	$^5H_3 \to {}^5D_4 = 690$
$^6G_{13/2} \to {}^6I_{17/2} = 676$	$^5F_4 \to {}^5D_4 = 674$
$^6G_{13/2} \to {}^6I_{9/2} = 665$	$^5F_3 \to {}^5D_4 = 624$
$^6G_{13/2} \to {}^6I_{7/2} = 650$	$^5I_7 \to {}^5D_4 = 618$
$^6G_{3/2} \to {}^6I_{9/2} = 704$	$^5F_2 \to {}^5D_4 = 601$
$^6G_{3/2} \to {}^6I_{7/2} = 686$	$^5F_1 \to {}^5D_4 = 587$
$^6G_{3/2} \to {}^6P_{3/2} = 583$	$^5I_4 \to {}^5D_4 = 587$
$^6G_{3/2} \to {}^6P_{5/2} = 565$	$^5I_6 \to {}^5D_4 = 582$
$^6G_{5/2} \to {}^6P_{3/2} = 609$	$^5I_5 \to {}^5D_4 = 570$
$^6G_{5/2} \to {}^6P_{5/2} = 589$	$^5K_8 \to {}^5D_4 = 495$
$^6G_{5/2} \to {}^6P_{7/2} = 571$	$^5G_6 \to {}^5D_4 = 487$
$^6G_{11/2} \to {}^6P_{3/2} = 611$	$^5D_3 \to {}^7F_5 = 413$
$^6G_{11/2} \to {}^6P_{5/2} = 592$	$^5D_3 \to {}^7F_4 = 435$
$^6G_{9/2} \to {}^6P_{3/2} = 613$	$^5D_3 \to {}^7F_3 = 455$
$^6G_{5/2} \to {}^6P_{5/2} = 593$	$^5D_3 \to {}^7F_2 = 469$
$^6G_{5/2} \to {}^6P_{7/2} = 575$	$^5D_3 \to {}^7F_0 = 485$
$(^6G,^6F)_{7/2} \to {}^6P_{3/2} = 626$	$^5D_4 \to {}^7F_6 = 488$
$(^6G,^6F)_{7/2} \to {}^6P_{5/2} = 605$	$^5D_4 \to {}^7F_5 = 543$
$(^6G,^6F)_{7/2} \to {}^6P_{7/2} = 586$	$^5D_4 \to {}^7F_4 = 582$
	$^5D_4 \to {}^7F_3 = 617$
	$^5D_4 \to {}^7F_2 = 644$
	$^5D_4 \to {}^7F_1 = 664$
	$^5D_4 \to {}^7F_0 = 674$

rapidly with increasing P_{ex}. As indicated in Fig. S6(a), the population density of the level E_2 can be expressed as $N_2 \propto P^2N_1$, where P is the excitation power and N_1 is the population density of the level E_1. Basically, we can divide N_2 into the radiative part N_2^r and the nonradiative part N_2^{nr}, i.e., $N_2 = N_2^r + N_2^{nr}$. The increase of the nonradiative decay rate or equivalently the decrease of the radiative decay rate with increasing P_{ex} can be expressed as $N_2^r/N_2 \propto P^{-x}$ $(x > 0)$, as shown in Fig. S6(b). Hence, the excitation power dependence of the luminescence intensity originating from the radiative transition from the level E_2 to the level E_3 can be written as $N_2^r \propto N_2P^{-x} \propto P^{2-x}N_1$ $(x > 0)$. If we plot the luminescence intensity as a function of the P_{ex} in a logarithmic coordinate, a slope of 2-x is observed, as schematically shown in Fig. S6(c). If $x > 1$, then a slope smaller than 1.0 is obtained. Therefore, the existence of P_{ex}-dependent nonradiative decay rate for the energy levels with energy separation comparable to the energy of phonons will lead to slopes much smaller than 2.0 in the two-photon-induced cascade emission. For the energy levels whose decays are governed by radiative one, such as the level $^5D_4$ in Tb^{3+}, slopes close to 2.0 are observed for the emission bands related to the level $^5D_4$, as shown in Fig. 4, Fig. S1 and Fig. S3.

3. Emissions between energy levels of Gd^{3+} and Tb^{3+} revealed in the two-photon-induced cascade emission

When the level ^6G_J in Gd^{3+} is effectively populated by resonant two-photon-induced absorption, the large number of electrons accumulated in the level ^6G_J makes the radiative transitions from the level ^6G_J to the levels ^6I_J and ^6P_J possible, leading to the emission of the first photons (see Fig. 1). After the energy transfer of electrons from Gd^{3+} to Tb^{3+}, the radiative transitions from the upper levels of Tb^{3+} to the levels $^5D_3$ and $^5D_4$ give rise to the second photons. During the photon cascade emission process, many emission bands which are invisible in the conventional excitation scheme become revealed in the emission spectrum, as shown in Fig. 6. In Table I, we summarize the electronic transitions related to the new emission bands originating from the two-photon-induced cascade emission [7,8].

4. Energy transfer from Gd^{3+} to Tb^{3+}

When the levels ^6G_J of Gd^{3+} are excited, the generated electrons will relax to the low-energy levels of ^6D_J, ^6I_J, and ^6P_J either radiatively or nonradiatively. Once the electrons populate the levels ^6D_J, ^6I_J, and ^6P_J, an energy transfer (ET) from Gd^{3+} to Tb^{3+} may occur via phonon-assisted dipole-dipole transition. Since the lifetimes of the electrons on the levels ^6D_J and ^6I_J are much shorter than that on the levels ^6P_J, the ET is mainly completed through the levels ^6P_J and it has been reported in many literatures (see Refs. 46-50 in the main text). In the previous works, Gd^{3+} was generally excited to the levels ^6I_J or ^6P_J by using single photon excitation at a wavelength of 275 or 312 nm. In our case, Gd^{3+} was excited to the levels ^6G_J by using a femtosecond (fs) laser at 400 nm through TPA, generating a large number of electrons on the levels ^6G_J. Since there exist some energy levels in Tb^{3+} whose energies are close to those of the levels ^6D_J and ^6I_J (see Table II for the details), it is possible that some electrons may have chance to transfer from Gd^{3+} to Tb^{3+} through the levels ^6D_J or ^6I_J and relax to the level $^5D_3$ (or $^5D_4$) of Tb^{3+}, giving rise to the second photon emission, as schematically shown in Fig. 1. As can be seen Fig. 6, some new emission peaks emerge in the spectra obtained by resonantly exciting the levels ^6G_J with fs laser pulses at 400 or 405 nm, such as the peaks located at 496, 564, 601, and 694

TABLE II. Comparison of the wavenumbers of the levels ^6D_J in Gd^{3+} with those of some energy levels in Tb^{3+}.

$Gd^{3+}(cm^{-1})$		$Tb^{3+}(cm^{-1})$	
$^6D_{9/2}$	39799	$(^3P,^5D)_2$	39548
$^6D_{1/2}$	40462	$^3I_6$	40114
$^6D_{7/2}$	40712	$^5K_8$	40749
$^6D_{3/2}$	40851	$^5G_6$	41082
$^6D_{5/2}$	40977	$(^3K,^3I)_5$	41236

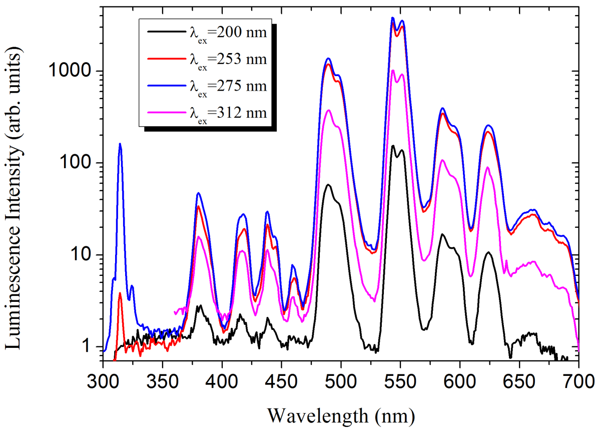

FIG. S7. Comparison of the emission spectra of the glass ($56SiO_2$-$10Al_2O_3$-$12Li_2O$-$20Gd_2O_3$-$2Tb_2O_3$ (mol%)) obtained by using λ_{ex} of 200, 253, 275, and 312 nm.

nm. These peaks are not observed when the levels ${}^6G_J$ are not efficiently populated by using an λ_{ex} at 390 nm. We think this is one of the evidences for the ET from Gd^{3+} to Tb^{3+} through the levels ${}^6D_J$. Another evidence for such ET is reflected in the emission spectra of the glass under single photon excitation with different λ_{ex}, as shown in Fig. S7. We excited the glass with λ_{ex} of 200, 253, 275, and 312 nm which are resonant with the levels ${}^6G_J$, ${}^6D_J$, ${}^6I_J$ and ${}^6P_J$ respectively and monitored the emission from the ${}^6P_J$ to ${}^8S_{7/2}$ transition in Gd^{3+}, which appears at 312 nm. The emission at 312 nm was not observed when we excited the glass at 200 nm, implying that most of electrons have been transferred to Tb^{3+} during the relaxation process. In contrast, we could observe the emission at 312 nm when exciting the glass at 253 nm and the emission intensity became much stronger when an λ_{ex} of 275 nm was used. This behavior indicates that some electrons recombine radiatively in Gd^{3+} and the efficiency of ET becomes smaller when exciting the glass at low-energy levels.

5. Relationship between the concentration of Gd^{3+}/Tb^{3+} and the ET efficiency

Basically, the ET from Gd^{3+} to Tb^{3+} is realized by two processes, including the energy migration between Gd^{3+} ions to a Gd^{3+} ion adjacent to a Tb^{3+} ion and then the ET from the Gd^{3+} ion to the Tb^{3+} ion [9]. Therefore, the ET efficiency depends strongly on the concentration of both Gd^{3+} and Tb^{3+} ions because it is proportional to the inverse sixth power of the distance between the two types of ions.

Previous studies showed that the emission intensity from Gd^{3+} at 312 nm decreased with increasing the concentration of Gd^{3+} when the concentration of Tb^{3+} was fixed, implying an increase in the ET efficiency with increasing the concentration of Gd^{3+} (see Ref. 47 in the main text and Ref. 9 in the Supplementary Information). In addition, an enhanced ET efficiency was also observed when the concentration of Tb^{3+} was increased (see Refs. 47, 48 and 50 in the main text). In our case, we measured the emission and excitation spectra of three glass samples with different concentrations of Gd^{3+}, as shown in Table III. The concentration of Gd^{3+} or Tb^{3+} was characterized by the ratio of Gd^{3+} or Tb^{3+} to the total host cations. While the properties of the sample I was described in detail in the main text, the measurement results of the samples II and III were provided in the Supplementary Information. In Table III, we provided the ratio of the emission intensity at 544 nm (emission from Tb^{3+}) to that at 312 nm (emission from Gd^{3+}) under the single photon excitation at 275 nm, which characterizes the ET efficiency from Gd^{3+} to Tb^{3+}. It can be seen that the ET efficiency decreased rapidly when the concentration of Gd^{3+} was reduced from 27.8% to 3.81%. In Fig. S8, we compare the excitation spectra measured for the three glass samples which have been normalized at the monitoring wavelength of 544 nm. It can be clearly seen that the excitation efficiencies for the levels ${}^6D_J$ and ${}^6I_J$ in Gd^{3+} dropped with decreasing the concentration of Gd^{3+}. We also compare the normalized emission spectra of the three glass samples obtained by using λ_{ex} = 275

TABLE III. Concentrations of Gd^{3+} and Tb^{3+} in the three glass samples used in our experiments. The ratio of the emission intensity at 544 nm to that at 312 nm in the emission spectra obtained by using λ_{ex} = 275 nm was also provided. The compositions (mol%) of the samples I, II and III are $56SiO_2$-$10Al_2O_3$-$12Li_2O$-$20Gd_2O_3$-$2Tb_2O_3$, $65SiO_2$-$13BaO$-$5K_2O$-$5Na_2O$-$1Al_2O_3$-$5Li_2O$-$5Gd_2O_3$-$1Tb_2O_3$, and $42.5CaO$-$15Al_2O_3$-$39B_2O_3$-$3Gd_2O_3$-$0.5Tb_2O_3$, respectively.

sample	Gd^{3+}	Tb^{3+}	ratio
I	27.8%	2.78%	25.3
II	8.2%	1.64%	1.1
III	3.81%	0.63%	0.4

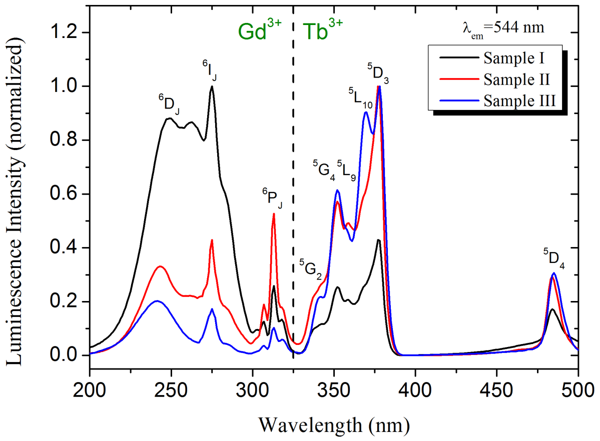

FIG. S8. Comparison of the excitation spectra of the three glass samples obtained by monitoring wavelength of 544 nm. The final energy states involved in the electronic transitions responsible for the excitation peaks of Gd^{3+} and Tb^{3+} are indicated in the left and right sides of the dashed line, respectively.

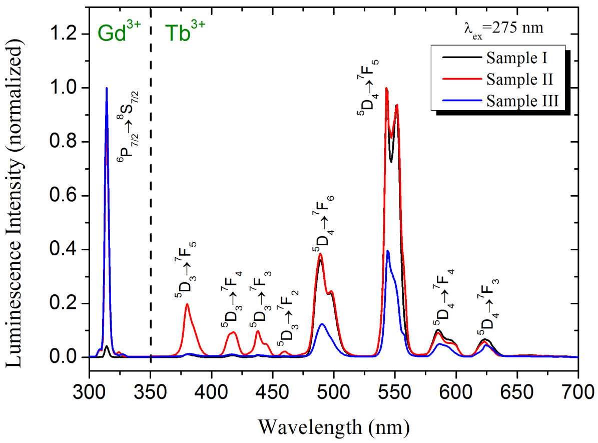

FIG. S9. Comparison of the emission spectra of the three glass samples obtained by using $\lambda_{ex} = 275$ nm. The electronic transitions responsible for the emission peaks of Gd^{3+} and Tb^{3+} are indicated in the left and right sides of the dashed line, respectively.

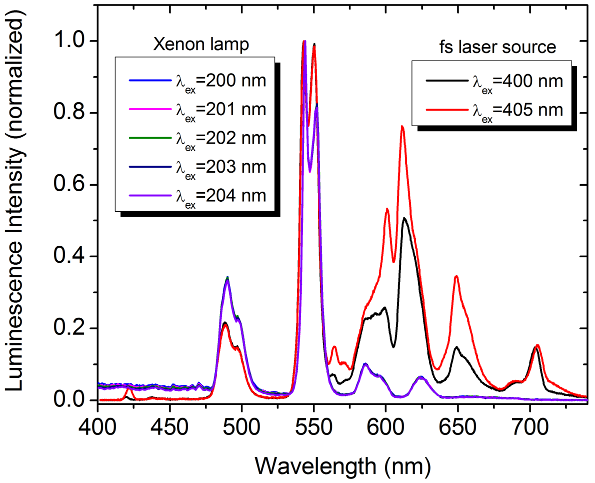

FIG. S10. Comparison of the luminescence spectra obtained by single-photon excitation and two-photon excitation for the silicate glass ($56SiO_2$-$10Al_2O_3$-$12Li_2O$-$20Gd_2O_3$-$2Tb_2O_3$ (mol%)).

nm, as shown in Fig. S9. For the sample I, the emission at 544 nm which corresponds to the $^5D_4 \rightarrow {}^7F_5$ transition in Tb^{3+} was about 23 times stronger than that at 312 nm which originates from the $^6P_{7/2} \rightarrow {}^8S_{7/2}$ transition in Gd^{3+}. It implies that most of the energy absorbed by the levels ^6I_J in Gd^{3+} has been transferred to Tb^{3+} in the sample I with a large concentration of Gd^{3+}. In sharp contrast, the ratio of the emission intensity at 544 nm to that at 312 nm was reduced to 1.1 and 0.4 in the samples II and III with smaller concentrations of Gd^{3+}, respectively. It means that the ET efficiency was reduced by decreasing the concentration of Gd^{3+}, increasing significantly the emission from Gd^{3+}. Moreover, we compared the tunable range of the luminescence color by varying the λ_{ex} and P_{ex} for several glass samples with different concentrations of Gd^{3+}. It was found that the best performance was achieved in the sample I with the largest concentration of Gd^{3+}.

6. Comparison of two-photon-induced and single-photon-induced luminescence

Apart from the potential application in laser-induced color display, the two-photon-induced photon cascade emission proposed and demonstrated in this work enables the observation of some electronic transitions which are not visible in the conventional single-photon excitation, as indicated in Fig. 6 and summarized in Table I. We have examined the single-photon-induced luminescence of the silicate glass ($56SiO_2$-$10Al_2O_3$-$12Li_2O$-$20Gd_2O_3$-$2Tb_2O_3$ (mol%)) and compared it with the two-photon-induced luminescence, as shown in Fig. S7. The single-photon-induced luminescence spectra were measured by using a spectrometer (F-4600, Hitachi) at different λ_{ex} ranging from 200 to 204 nm and no obvious difference was found in the spectra. The spectral shape is quit similar to that of the glass ($45CaO$-$15Al_2O_3$-$39.5B_2O_3$-$0.5Tb_2O_3$ (mol%)) doped only with Tb^{3+}, as shown in Fig. S3. The new emissions originating from the two-photon-induced photon cascade emission are not observed. The physical origins responsible for this difference are twofold. First, there are many energy states in rare-earth ions which are inactive for single-photon excitation but active for two-photon excitation because of the conservation of party during the electric transitions. Such energy states may play a crucial role in the photon cascade emission followed by two-photon excitation. Second, the density of electrons generated by two-photon absorption induced by femtosecond laser with a high peak power is much larger than that generated by single-photon excitation. As a result, the number of electrons that recombine radiatively is increased. However, more experiments are needed to confirm these suspects.

7. Calculation of CIE chromaticity coordinate

Based on the theory of colorimetry, the color of a material can be represented by tristimulus values (X, Y, and Z) and the relationship between the tristimulus values and the chromaticity coordinates (x, y, and z) is described as follows [10]:

$$\begin{cases} X = k\int_\lambda S(\lambda)\overline{x}(\lambda)\beta(\lambda)d\lambda \\ Y = k\int_\lambda S(\lambda)\overline{y}(\lambda)\beta(\lambda)d\lambda \\ Z = k\int_\lambda S(\lambda)\overline{z}(\lambda)\beta(\lambda)d\lambda \\ x = \frac{X}{X} + Y + Z \\ y = \frac{Y}{X} + Y + Z \\ z = \frac{Z}{X} + Y + Z \end{cases}$$

Here, $S(\lambda)$ is the relative power of the illumination at wavelength λ, $\overline{x}(\lambda)$, $\overline{y}(\lambda)$, $\overline{z}(\lambda)$are the color matching functions for the CIE 1931 2° Standard Observer, $\beta(\lambda)$ is the spectral reflectivity of the sample at wavelength λ.

Based on the above equation and emission spectra of the glass under different λ_{ex} and P_{ex}, we could easily derive the evolution of the CIE chromaticity coordinate (x, y, and z) when λ_{ex} or P_{ex} was varied (see Figs. 5, S2 and S4).

---

[1] van Dijk, J. M. F. & Schuurmans, M. F. H. On the nonradiative and radiative decay rates and a modified exponential energy gap law for 4f-4f transitions in rare-earth ions. *J. Chem. Phys.* **78,** 5317-5323 (1983).

[2] Li, G. C. *et al.* Efficient three-photon luminescence with strong polarization dependence from a scintillating silicate glass co-doped with Gd^{3+} and Tb^{3+}. *Opt. Express* **21,** 6020-6027 (2013).

[3] Pollnau, M., Gamelin, D. R., Lthi, S. R., Gdel, H. U. & Hehlen, M. P. Power dependence of upconversion luminescence in lanthanide and transition-metal-ion systems. *Phys. Rev. B* **61,** 3337-3346 (2000).

[4] Yuan, M. H. *et al.* Efficient blue light emission from $In_{0.16}Ga_{0.84}N/GaN$ multiple quantum wells excited by 2.48-μm femtosecond laser pulses. *Opt. Lett.* **39,** 3555-3558 (2014).

[5] Yang, Z., Lin, J. H., Su, M. Z., Tao, Y. & Wang, W. Photon cascade luminescence of Gd^{3+} in $GdBaB_9O_{16}$. *J. Alloy. Comp.* **308,** 94-97 (2000).

[6] Zheng, K., Zhao, D., Zhang, D., Liu, N. & Qin, W. Temperature-dependent six-photon upconversion fluorescence of Er^{3+}. *J. Fluorine. Chem.* **132,** 5-8 (2011).

[7] Carnall, W. T., Fields, P. R. & Rajnak, K. Electronic energy levels of the trivalent lanthanide aquo ions. II. Gd^{3+}. *J. Chem. Phys.* **49,** 4443-4446 (1968).

[8] Carnall, W. T., Fields, P. R. & Rajnak, K. Electronic energy levels of the trivalent lanthanide aquo ions. III. Tb^{3+}. *J. Chem. Phys.* **49,** 4447-4449 (1968).

[9] Wang, S., Qian, Q., Zhang, Q. Y., Yang, Z. M. & Jiang, Z. H. Gd^{3+}-sensitized Tb^{3+}-doped scintillating silicate glasses. *J. Inorg. Mater.* **24,** 773-777 (2009).

[10] Bunting, F. *The Colorshop Color Primer*, 74-75 (Light Source Computer Images, Inc. An X-Rite Company, 1998).
